# Modeling the effects of hyaluronic acid degradation on the regulation of human astrocyte phenotype using multicomponent interpenetrating polymer networks (mIPNs)

**DOI:** 10.1038/s41598-020-77655-1

**Published:** 2020-11-26

**Authors:** Andrea C. Jimenez-Vergara, Rachel Van Drunen, Tyler Cagle, Dany J. Munoz-Pinto

**Affiliations:** 1 Engineering Science Department, Trinity University, San Antonio, TX 78212 USA; 2 Neuroscience Program, Trinity University, San Antonio, TX 78212 USA; 3 Department of Engineering Science, Neuroscience Program, Center for the Sciences and Innovation, CSI 470C, Trinity University, One Trinity Place, San Antonio, TX 78212 USA

**Keywords:** Biomedical engineering, Bioinspired materials, Astrocyte, Polymers

## Abstract

Hyaluronic acid (HA) is a highly abundant component in the extracellular matrix (ECM) and a fundamental element to the architecture and the physiology of the central nervous system (CNS). Often, HA degradation occurs when an overreactive inflammatory response, derived from tissue trauma or neurodegenerative diseases such as Alzheimer’s, causes the ECM in the CNS to be remodeled. Herein, we studied the effects of HA content as a key regulator of human astrocyte (HAf) reactivity using multicomponent interpenetrating polymer networks (mIPNs) comprised of Collagen I, HA and poly(ethylene glycol) diacrylate. The selected platform facilities the modulation of HA levels independently of matrix rigidity. Total astrocytic processes length, number of endpoints, the expression of the quiescent markers: Aldehyde Dehydrogenase 1 Family Member L1 (ALDH1L1) and Glutamate Aspartate Transporter (GLAST); the reactive markers: Glial Fibrillary Acidic Protein (GFAP) and S100 Calcium-Binding Protein β (S100β); and the inflammatory markers: Inducible Nitric Oxide Synthase (iNOS), Interleukin 1β (IL-1β) and Tumor Necrosis Factor Alpha (TNFα), were assessed. Cumulatively, our results demonstrated that the decrease in HA concentration elicited a reduction in the total length of astrocytic processes and an increase in the expression of HAf reactive and inflammatory markers.

## Introduction

Astrocytes are integral to the central nervous system (CNS) tissue^1^. They provide nutrients to neurons and support neural functions including axonal guidance, synaptic support, and maintenance of the blood brain barrier^2–6^. In vivo, astrocytes exhibit a star-like morphology with small cells bodies and radial branched processes^7,8^ whose tendrils extend to wrap around nearby neurons, providing structural support to the CNS tissue architecture. Furthermore, astrocytes are responsible for the deposition of extracellular matrix (ECM) components and the remodeling of the microenvironment surrounding CNS cells.

Once considered to play only a minor role as “helper cells” in the CNS, astrocytes have become the focus of numerous studies due to their role in the onset and progression of several neurodegenerative diseases such as Alzheimers’ disease (AD) and Parkinson’s disease. Moreover, astrocytes are key players during the inflammatory response and healing process of damaged nervous tissue. Astrocytes adopt a reactive phenotype in response to CNS tissue damage, trauma^9^, or the abnormal presence of ECM structures such as amyloid beta (Aβ) plaques and tau entanglements^10,11^. The transition from quiescent to reactive astrocytes can be characterized by monitoring changes in cell morphology and the relative expression of Glial Fibrillary Acidic Protein (GFAP), Aldehyde Dehydrogenase 1 Family Member L1 (ALDH1L1), Aldolase C (AldoC), Glutamate Aspartate Transporter (GLAST), and S100 Calcium-Binding Protein β (S100β) among others^12,13^.

The change from the quiescence state of astrocytes to a reactive phenotype propagates the degradation and removal of abnormal ECM components, dead cells, and cell debris facilitating healing by remodeling the microenvironment of the damaged tissue^1,14^. In the CNS, the microenvironment is characterized by a dynamic, delicate and bidirectional set of interactions between CNS cells and the ECM components. Macromolecules such as: Collagen Type IV (ColIV), hyaluronic acid (HA), and laminin characterize the ECM landscape of the CNS^15,16^. These main components contribute to the regulation of normal astrocyte function and the modulation of their phenotype^17–19^. Following tissue damage or cell death due to accumulation of Aβ or tissue trauma, the surrounding ECM is degraded by the enzymatic action of metalloproteases (MMPs) and hyaluronidases initially secreted by microglial cells^20,21^. During this process, the degradation of tissue modulates the relative content and identity of the main ECM components. Alterations of the ECM components combined with the release of ECM fragments increases the inflammatory response of microglia and contributes to the acquisition of a reactive astrocytic phenotype^22^. This process escalates the production and activity of MMPs and hyaluronidases creating a vicious cycle whereby the inflammatory response promotes the over-degradation of damaged tissue. Continuous degradation of the ECM alters its viscoelastic properties producing attenuated mechanical integrity and therefore a reduction in the microenvironment rigidity. On the other hand, scar tissue which is rich in collagen, fibronectin and laminin^23^ deposited by fibroblast or endothelial cells can augment tissue rigidity in the damaged area. Vicissitudes in ECM composition and the alterations to viscoelastic properties of damaged tissue are well known as significant modulatory factors of astrogliosis^24^.

To study key characteristics of the function and interactions between astrocytes and ECM components in the CNS, in vitro platforms have typically been established using 2D microenvironments. These 2D environments, however, do not accurately resemble the viscoelastic properties of CNS tissue nor do they capture the 3D cell milieu of native tissue. Moreover, findings from 2D studies cannot be easily extrapolated to the behavior of cells in 3D contexts^25^. In fact, the behavior of astrocytes and their impact on other CNS cells in 2D culture strongly diverges from that seen in a 3D culture^26^. Other studies have found that 2D culture platforms promote astrocyte activation^27^ whereas 3D culture platforms are capable of sustaining astrocytes in their quiescent state^8,28^.

To fabricate a 3D in vitro culture model to study the behavior of CNS cells, research efforts have focused on bioinspired hydrogels that recapitulate some of the key biochemical cues for the main ECM constituents in the CNS^29,30^. Both natural and synthetic materials have been utilized to mimic the 3D landscape of CNS tissue. For instance, Collagen Type I (ColI) has been used as the foundational component of engineered scaffolds due to its self-assembly properties, similar chemical composition to ColIV, and its capacity to promote the spreading and cell growth of CNS cells^8,31–33^. Given its high presence in CNS tissue, other approaches have employed HA-based materials for the fabrication of engineered scaffolds^8,34^. Natural based polymer scaffolds also provide great biochemical compatibility. However, natural polymers alone are not sufficient to withstand the physiological pressure induced by cellular proliferation, spreading, and cell-mediated degradation^35,36^.

To address this challenge, we used a multicomponent interpenetrating polymer network (mIPN) system comprised of ColI, HA, and poly(ethylene glycol) diacrylate (PEGDA). The mIPN fabrication, mechanical characterization and initial evaluation of their cytocompatibility (≥ 84%) for cells in CNS was previously described by Van Drunen et al.^33^. ColI and HA were employed to promote cell attachment, spreading, and ECM mediated signaling. Linear PEGDA was used to tune the mechanical performance of the engineered scaffold to closely match the complex modulus of mouse brain cortex tissue (≈ 6.9 kPa) and to maintain the physical integrity of the scaffolds^33^.

Herein our research team utilized this scaffold platform to investigate the effects of HA degradation on the regulation of human astrocyte phenotype independently of matrix rigidity. As CNS tissue degrades by MMPs and hyaluronidases, the content of HA high molecular weight (HA-HMW) decreases^37,38^. In this work, we evaluated the changes in astrocyte phenotype at the gene and protein expression levels as a function of the decrease in HA-HMW content. Astrocyte responses to different HA-HMW concentration were monitored in terms of cell spreading; the expression of the quiescent markers: ALDH1L1, and GLAST; the reactive makers GFAP, and S100β; and the inflammatory markers interleukin 1β (IL-1β), inducible nitric oxide synthase (iNOS), and tumor necrosis factor alpha (TNFα). In addition, the expression of the Homing Cell Adhesion Molecule (HCAM or CD44), a cell receptor for HA was evaluated at the protein expression level. Three mIPN formulations containing 2 mg/mL, 1 mg/mL, and 0 mg/mL of HA-HMW and exhibiting a complex modulus between 6.4 kPa and 7.4 kPa were employed (Table1).

**Table 1:** Composition and complex modulus of mIPNs.

mIPN	ColI (mg/mL)	HA (mg/mL)	PEGDA molecular weight (kDa)	PEGDA final concentration (%w/w)	Complex modulus (kPa)
0 mg/mL HA	3.0	0.0	10.0	7.0	7.4 ± 0.3
1 mg/mL HA	3.0	1.0	3.4	7.0	6.8 ± 1.9
2 mg/mL HA	3.0	2.0	3.4	7.5	6.4 ± 0.9

In this work, we demonstrated that the reduction in HA-HMW content in the proposed scaffolds attenuates astrocyte spreading and the quantity of cell processes. In addition, the expression of the selected quiescent markers decreases as HA-HMW is reduced in the engineered matrix. Furthermore, the expression of the reactive phenotype markers and inflammatory cytokines increases in the low HA-HMW containing mIPNs. We demonstrated that the reduction in HA-HMW concentration—which is associated with HA degradation—is a significant contributing factor to the modulation of human astrocyte phenotype. Specifically, the reduction of HA-HMW promotes astrocyte reactivity and their inflammatory responses.

## Materials and methods

### PEGDA and photoinitiator synthesis

Linear PEG (3.4 or 10.0 kDa, Sigma) was reacted in anhydrous dichloromethane (Sigma) with acryloyl chloride (Sigma) using 4:1 (3.4 kDa) or 8:1 (10.0 kDa) molar ratio relative to PEG. Trimethylamine (Sigma) was added to the reaction mixture using a 2:1 molar ratio. The reaction was carried out at 4 °C and the reaction product was purified as previously described^39^. The substitution level of hydroxyl groups was confirmed to reach ≈ 95% by ^1^H NMR.

Lithium phenyl-2,4,6-trimethylbenzoylphosphinate (LAP) was selected as the photoinitiator for the PEGDA polymerization. This photoinitiator was synthesized as previously described by Fairbanks et al.^40^. In brief, 3.0 g of dimethyl phenylphosphonite (Alfa Aesar) were mixed with 3.2 g of 2,4,6-trimethylbenzoyl chloride (Alfa Aesar) under nitrogen. After 18 h of stirring at room temperature the reaction mixture was mixed with 6.1 g of lithium bromide (Acros Organics) in 100 mL of 2-butanone (Fisher). The resulting solution was heated at 50 °C for 10 min and allowed to rest for four hours at room temperature. The formed solid was filtered, rinsed with 2-butanone under vacuum three times and dried under vacuum. LAP was characterized by ^1^H NMR. The resulting LAP was dissolved in water to a concentration of 100 mM.

### Cells encapsulation

Primary human astrocytes (HAf, Cell Applications) at passage 4 were expanded in monolayer and cultured in DMEM/F12 50:50 (Corning) medium supplemented with 10% FBS (Atlanta biologicals), 1% Glutamax (Gibco) and 1% PS (Gibco). Cells were maintained at 37 °C and 5% CO_2_ prior to encapsulation.

**Figure 1:**
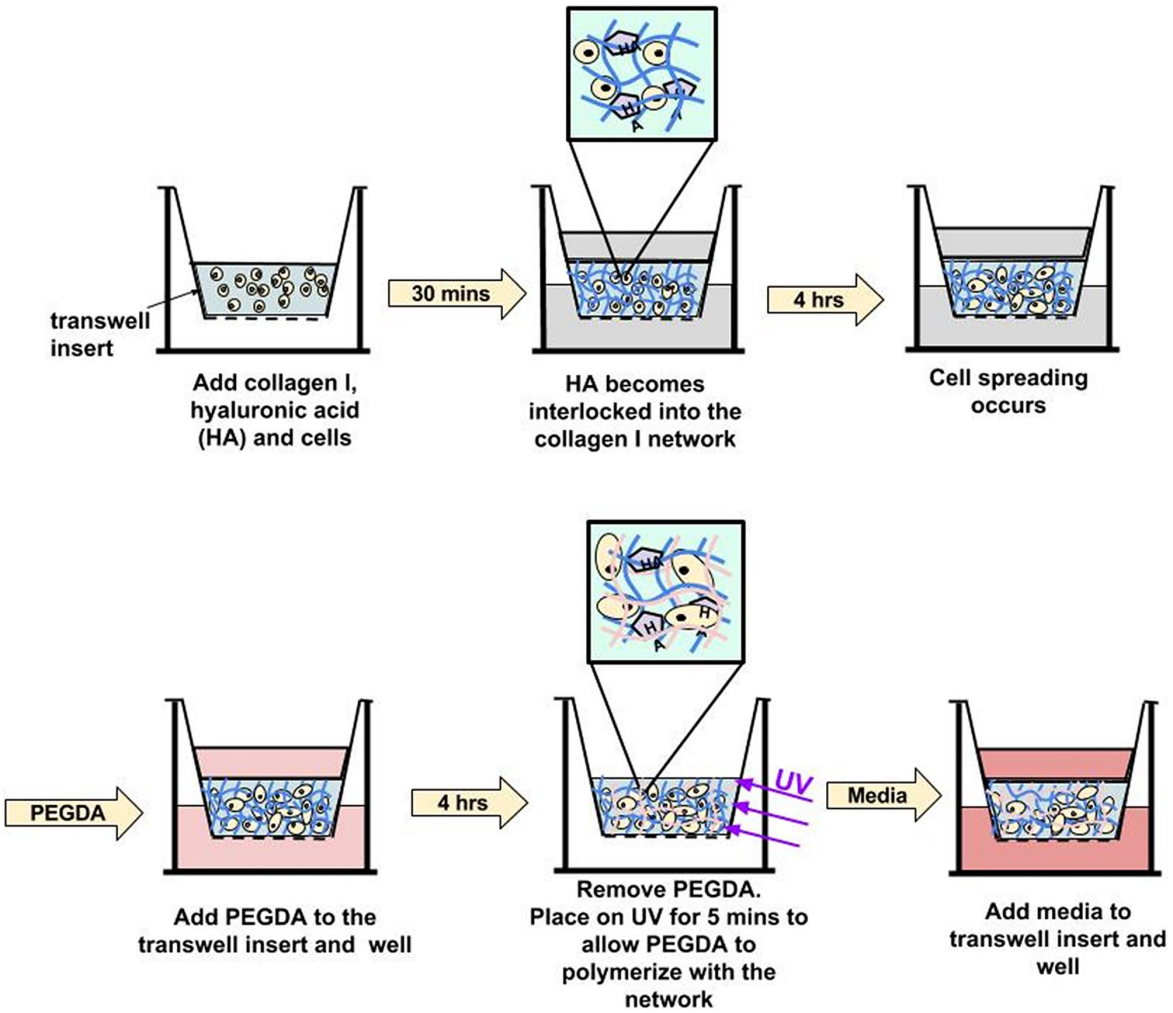
Schematic representation of the fabrication of collagen-HA-PEGDA mIPN hydrogels.

At passage 5, HAf were harvested and encapsulated at 8.0 × 10^5^ cells/mL in mIPN formulations containing constant levels of ColI (3 mg/mL) and 0, 1 or 2 mg/mL of HA-HMW and appropriate levels of 3.4 kDa or 10.0 kDa PEGDA (Fig. 1) to achieve materials with similar complex modulus (Table 1). The mIPN formulations were selected based on the mechanical properties previously screened and published by our laboratory^33^. In brief, HA-HMW (Lifecore Biomedical, 1.35–1.38 MDa) at the desired concentration was dissolved in an aqueous neutralization solution containing NaOH and 10X PBS. Ice-cold high-concentration rat tail ColI (BD Bioscences, 9.66 mg/mL in 0.02 N acetic acid) was then diluted to 3 mg/mL in the cold neutralization solution. The HAf were resuspended in the neutralized ice-cold solution. Three hundred microliters of the cell suspension were then pipetted into BD Falcon culture inserts (12 mm diameter, 8 μm pore size). The constructs were then incubated for 30 min at 37 °C, 5% CO_2_ and 95% relative humidity. During this incubation period, the collagen was allowed to crosslink resulting in the formation of the first polymer network containing physically entrapped HA-HMW molecules. The resulting ColI-HA gels were then immersed in phenol-red free DMEM/F12 50:50 (Hyclone) supplemented with 10% FBS, 1% PS and HA-HMW at the same concentration as in the ColI-HA gels. Cells were allowed to spread for 4 h in the ColI-HA constructs prior to the immersion of the constructs in the corresponding PEGDA precursor solution. After 4 h, the media was carefully replaced with 1.7 mL of sterile-filtered PEGDA precursor solution in phenol-red free DMEM/F12 50:50 supplemented with 5% FBS, 1% PS, HA-HMW and, 1% v/v of LAP as reported in (Table 1). The PEGDA solution was allowed to permeate the structure of the ColI-HA gel for 4 h while incubated at 37 °C with 5% CO_2_. Afterwards, the excess of PEGDA solution was removed. The constructs were then exposed to long wave UV for 5 min (Spectroline, = 6 mW/cm^2^, 365 nm). The selected mIPNs formulations contain different concentrations of HA-HMW while maintaining similar complex modulus within the range of the elastic properties of brain cortex tissue^33,41,42^.

For each mIPN formulation, the resulting hydrogels were divided in three groups: 0, 3 and 10 days. The mIPNs were cultured in DMEM/F12 supplemented with 10% FBS and 1% PS at 37 °C, 5% CO_2_ and 95% relative humidity. Six independent samples per group were collected at each corresponding time point. Zero-day samples were fixed with 10% buffered formalin and stored in the fixative solution at 4 °C until use. The zero-day samples were used for the spreading analysis. Day 3 and day 10 constructs were split in two during the sample collection. One part from each sample was fixed and stored in the fixative solution at 4 °C until use. The fixed samples from day 3 and day 10 were used for cytological analyses. The other half from each specimen was snap-frozen and stored at − 80 °C for gene expression assessments.

### Cell viability

The fabrication process of the mIPNs involves the use of UV radiation during the polymerization of the PEGDA. Therefore, to assess the effects of the fabrication process on cell viability, the Live/Dead assay (Invitrogen, Life Technologies) was performed 24 h post hydrogel fabrication. Samples were collected and images from samples specimens were taken following the process described by Munoz-Pinto et al.^36^.

### Hydrogel mechanical characterization

The viscoelastic properties of the mPINs were measured by dynamic mechanical analysis (DMA) using a TA Instruments Electroforce 3100 mechanical tester. Four independent specimens per mIPN formulation of approximately 8 mm in diameter and 1 mm in height were preloaded with a 2 g force and exposed to oscillations of 100 μm in amplitude at 1 Hz of frequency. The storage (Eʹ) and loss (Eʺ) moduli were recorded and the complex modulus (E^*^) calculated as $E^{*} = \sqrt {E^{{\prime}{2}} + E^{{\prime\prime}{2}} }$.

### Cell morphology measurements

#### Confocal microscopy imaging

Approximately 1 mm transverse sections from the 0 day time point were cut manually and fixed with formalin. The sample slices were washed with PBS 3 times and stained with Rhodamine Phalloidin (Life technologies, 1:100) and DAPI dilactate (4′,6-diamidino-2-phenylindole, Life technologies, 300 nM). Following overnight incubation at 4 °C, the slices were washed with PBS three times and placed in a 35 mm glass bottom dish surrounded by a small amount of PBS to prevent dehydration during the imaging process. Images were acquired using a Nikon A1 confocal microscope system equipped with a 20X objective. Three regions per sample were randomly selected for imaging and six to seven consecutive images in the z direction were taken per region.

#### Cell morphology analysis

The total branch length and the number of endpoints were assessed using ImageJ2 (Fiji) and a modified protocol developed by Young et al.^43^. In brief, 6 to 7 consecutive images with an optical thickness of 25 μm from each region in the hydrogel were stacked to generate z-stack projection image. A skeleton was generated from the stack projection. Branch length and number of endpoints were calculated using the plugin AnalyzeSkeleton (2D/3D). To illustrate this analysis, a raw image of a cell and its skeleton projection highlighting the cell center and branch length have been included in the Supplementary Fig. 1. Four independent specimens per experimental group containing between 50 and 130 cells in each 3D projection were analyzed.

### Extraction of mRNA and gene expression evaluation

The astrocyte phenotype was evaluated after 3 and 10 days in culture using qRT-PCR. For each time point, the relative expression of each selected maker was normalized relative to the 0 mg/mL HA mIPN group. The mRNA from 4–6 independent samples per group was extracted using the Dynabeads mRNA direct kit (Ambion, Life Technologies) as described by Jimenez-Vergara et al.^44^. The purified polyA-mRNA was stored at − 80 °C until use.

Gene expression evaluation of HAf was performed using a 7500 Real-Time PCR Systems (Applied Biosystems) and the SuperScript III Platinum One-Step qRT-PCR kit (Invitrogen, Life Technologies). The mRNA levels for ALDH1L1, GLAST, GFAP, S100β, iNOS, IL-1β and tumor necrosis factor alpha TNFα genes were assessed in duplicate for each construct. Primer sequences are shown in Supplementary Table 1. Approximately 15 ng of polyA-mRNA and 5 µL of 1 mM primer were added per 25 µL of reaction mixture. Changes in SYBR Green fluorescence was used to monitor each reaction amplification. In addition, ROX dye was used as a passive reference. For each sample the gene expression relative to the reference gene (β-actin) was calculated using the ΔΔCt method. The appropriate amplification product for each PCR reaction was verified by using the melting temperature curve.

### Protein expression assessments

Protein expression of the markers ALDH1L1, GLAST, GFAP, S100β iNOS, IL-1β, TNFα and HCAM was assessed by immunostaining. Transverse section of approximately 1 mm in thickness were cut from each formalin fixed sample. Hydrogel sections were then blocked with PBST (PBS plus 0.1% Tween 20) containing 3% BSA for 12 h at 4 °C. Primary antibody (Supplementary Table 2) was diluted in PBS containing 3% BSA and then applied to the sections overnight at 4 °C. After rinsing with PBS, the sections were exposed to Alexa 488 anti-Mouse (Life Technologies) and DAPI dilactate for 12 h at 4 °C. Following rinsing in PBS to remove unbound secondary antibody, confocal microscopy was performed using a confocal microscope equipped with a 20X objective. Two randomly selected regions in each sample segment were imaged. A total of 5 to 16 independent images from 4 specimens per formulation were taken using an optical thickness of 50 μm. Between 40 and 260 cells per image were analyzed.

Semi-quantitatively evaluation of immunostaining results was conducted by counting the number of cells stained with Alexa 488 antibody and the number of cells stained with DAPI (total cell number). Cell counting was performed from each image using ImageJ software^36^. For each staining, the laser power, detector gain, and offset were maintained constant across all imaged specimens and sample groups. Fluorescence intensity for each stained image was quantified using ImageJ. Immunostaining intensity (d) for each image was determined using the equation:

$$d=\frac{\mathrm{number\,of\,cells\,stained \times Fluoresce\,intensity}}{\mathrm{total\,cell\,number}}.$$

### Statistical analysis

Data are reported as mean ± standard deviation. Comparison of sample means was performed using ANOVA followed by Tukey's post-hoc test (SPSS software). The mean and standard deviation was computed from at least four independent samples per experimental group. A p-value of 0.05 or less was considered statistically significant.

## Results and discussion

### Cell viability

The total cell viability for each formulation was estimated by taking the ratio between living cells and total cell count revealed that 24 h post fabrication about 94.0 ± 2.2%, 93.2 ± 1.5% and 94.8 ± 0.7% of cells were alive in the 2 mg/mL, 1 mg/mL and 0 mg/mL HA mIPN formulations respectively (Supplementary Fig. 2). Differences across formulations were not statistically significant (p ≥ 0.504). These viability results are similar to those observed for fibroblast, human adult stem cells, and CNS cells using PEGDA-based hydrogels and the UV polymerization system^33,40,45^.

### Hydrogel mechanical characterization

Viscoelastic properties play an important regulatory role in the progression and adoption of specific cell phenotypes^46^. Therefore, to fabricate a 3D model of CNS tissue it is important that the mechanical performance displayed by engineered scaffolds is similar to that in native tissue. Cells in the CNS tissue experience a microenvironment characterized by low rigidity values^47^. In this work, the selected mIPN system allowed us to tune the viscoelastic properties of the proposed biomaterials to be in close proximity to the mechanical behavior of mouse brain cortex tissue (≈ 6.9 kPa)^33^. Biochemical cues in the mIPNs were provided by the presence of constant levels of ColI (3 mg/mL) and varying levels of HA (2 mg/mL, 1 mg/mL and 0 mg/mL). The complex modulus of the constructs was maintained within the vicinity of that in mouse brain cortex tissue by the use of 3.4 kDa PEGDA and 10 kDa PEGDA at 7.0% and 7.5% w/w concentration. As shown in Table 1, the different selected mIPNs do not display significant differences in the complex modulus (p ≥ 0.646) and their performance is similar to brain cortex previously reported in literature^33,41,42^.

### Cell morphology measurements

**Figure 2:**
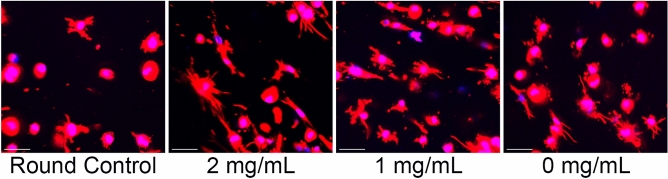
Representative images of HAf spreading in mIPNs containing distinct levels of HA. The scale bar represents a distance of 50 µm.

Achieving physiological cell morphology is one main challenge in culturing astrocytes for brain research^8^. Cell shape is a key physiological feature that contributes to normal cell function and overall tissue physiology. The proposed family of mIPNs promotes the adoption of star-shaped morphology in human astrocytes in a 3D context. While the mechanical performance in the scaffolds was consistent across each formulation, the compositions varied in terms of the HA content. To evaluate the effects of HA concentration in the mIPNs on astrocyte cell morphology, HAf were allowed to spread in each formulation for 4 h before starting the diffusion of PEGDA. The 4 h spreading time was identified as the maximum time the cells can be allowed to spread without significant cell-mediated bulk matrix contraction. The spreading time depends on cell type and matrix composition. Other studies using a similar systems including one by Munoz-Pinto et al. identified 6 h as an appropriate time frame to promote the spreading of human mesenchymal stem cells (hMSCs) while preventing cell mediated contraction of the scaffold^36^. Furthermore, Khetan et al. determined that 7 days in culture was sufficient to promote the spreading of hMSCs in a HA-based matrix^48^. To highlight the changes in cell morphology as a function of time, a round control group was included. In the round control group, HAf were allowed to spread for just 30 min in a 0 mg/mL HA mIPN before the PEGDA infiltration and its subsequent photopolymerization. Cell morphology was then evaluated in terms of total branch length and number of endpoints (Fig. 2).

**Figure 3:**
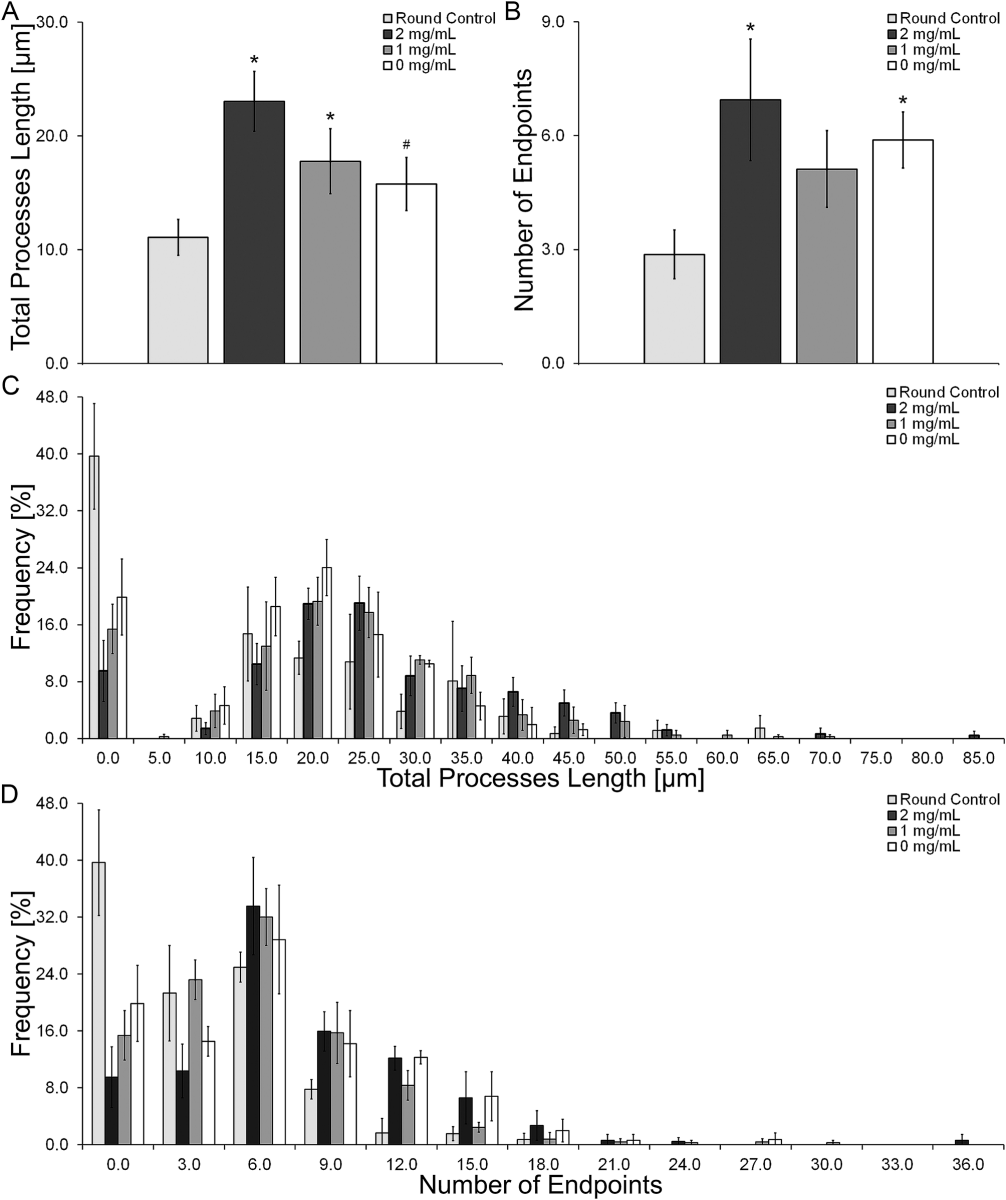
Average total processes length (**A**), average number of endpoints (**B**), Total processes length distribution (**C**) and number of endpoint distribution (**D**). *Significantly different from round control; ^#^Significantly different from 2 mg/mL HA. All reported significant differences were reported for p < 0.05.

The results from the morphology analysis indicate an overall increase in total branch length and the number of endpoints after 4 h of spreading relative to round control group (Figs. 2, 3A,B). Most of the astrocytes in the control group showed round morphologies (Fig. 2) while the astrocytes that were allowed to spread for 4 h exhibited the characteristic star shaped morphologies (Fig. 2). The total processes length was increased by 2.1-fold in the 2 mg/mL HA mIPN, 1.6-fold in the 1 mg/mL HA mIPN and 1.4-fold in the 0 mg/mL HA mIPN relative to the round control group. However, the increase in total branch length (Fig. 3A) was only significantly different from the control group in the 2 mg/mL (p = 0.001) and 1 mg/mL (p = 0.037) mIPNs. In addition, the total branch length appears to decrease with the reduction in HA content. The average processes length decreases from 23.1 µm in the 2 mg/mL HA to 17.8 µm for 1 mg/mL HA (p = 0.103) and to 15.8 µm for 0 mg/mL HA (p = 0.025) mIPN. Furthermore, it is important to highlight that the distribution curves for branch length of HAf allowed to spread for 4 h exhibited similar shape and dispersion behavior across experimental groups. The maximum average processes length (Fig. 3C) measured in at least one cell per image. In the 2 mg/mL mIPN the maximum was 80.4 µm while the maximum average processes length in the 1 mg/mL and 0 mg/mL mIPNs were 66.8 µm and 53.2 µm respectively. As expected the distribution in the round control group was shifted toward the left of the figure indicating low levels of cell spreading and round morphologies. The measured cell dimensions are in close agreement and within the range of recently published results for the morphological characterization of human astrocytes in the PEG based hydrogels^49^.

Relative to the round control group, there was an increase in the total number of endpoints of 2.4-fold in the 2 mg/mL HA mIPN, 1.2-fold in the 1 mg/mL HA mIPN and 1.4-fold in the 0 HA mg/mL mIPN (Fig. 3B). Significant differences in the total number of endpoints from the round control group were identified for the 2 mg/mL HA mIPN (p = 0.007) and 0 mg/mL HA mIPN (p = 0.035). Analysis of the number of endpoints reveal an apparent decrease for the 1 mg/mL HA and 0 HA mg/mL relative to 2 mg/mL HA mIPN, but statistical differences were not observed (p ≥ 0.634). The distribution curves for the number of endpoints shows similar behavior as the distribution for the total branch length. Figure 3D reveals that the maximum number endpoints in HAf allowed to spread for 4 h were 34 for the 2 mg/mL HA mIPN, 29 for the 1 mg/mL HA mIPN and 27 for the 0 mg/mL HA mIPN. Cumulatively, the morphology analysis reveals that relative to the round control group, the selected 4 h spreading time is sufficient to achieve significant differences in average branching length and total number of endpoints in HAf (Fig. 3A,B). Moreover, the total branch length and total number of endpoints is modulated by the reduction in HA content in the mIPNs (Fig. 3A,B). In human brain tissue, it has been reported that the maximum average processes length and number of branches per cell exhibited by protoplasmic astrocytes is approximately 98 μm in length and approximately 37 branches per astrocyte^50^. In our mIPN model human astrocytes reach approximately 25% of the average values for processes length and average number of endpoints. In our platform, the reduction in cell spreading with the decrease in HA content in the mIPNs agrees with previously reported results from Placone et al.^8^. However, in Placone’s work the change in astrocyte shape was linked to both HA content and scaffold stiffness. Our study improves on previously reported literature since our proposed mIPN system allows for the study of the effects of HA content on human astrocyte shape independently from the impact of scaffold stiffness. In our 3D in vitro model, the reduction in HA content recapitulated the reduction of HA in natural tissue as a characteristic change during tissue injury. This model shows that the reduction in HA levels contributes to the loss of the characteristic and physiological morphology of human astrocytes.

### Gene and protein expression analyses

**Figure 4:**
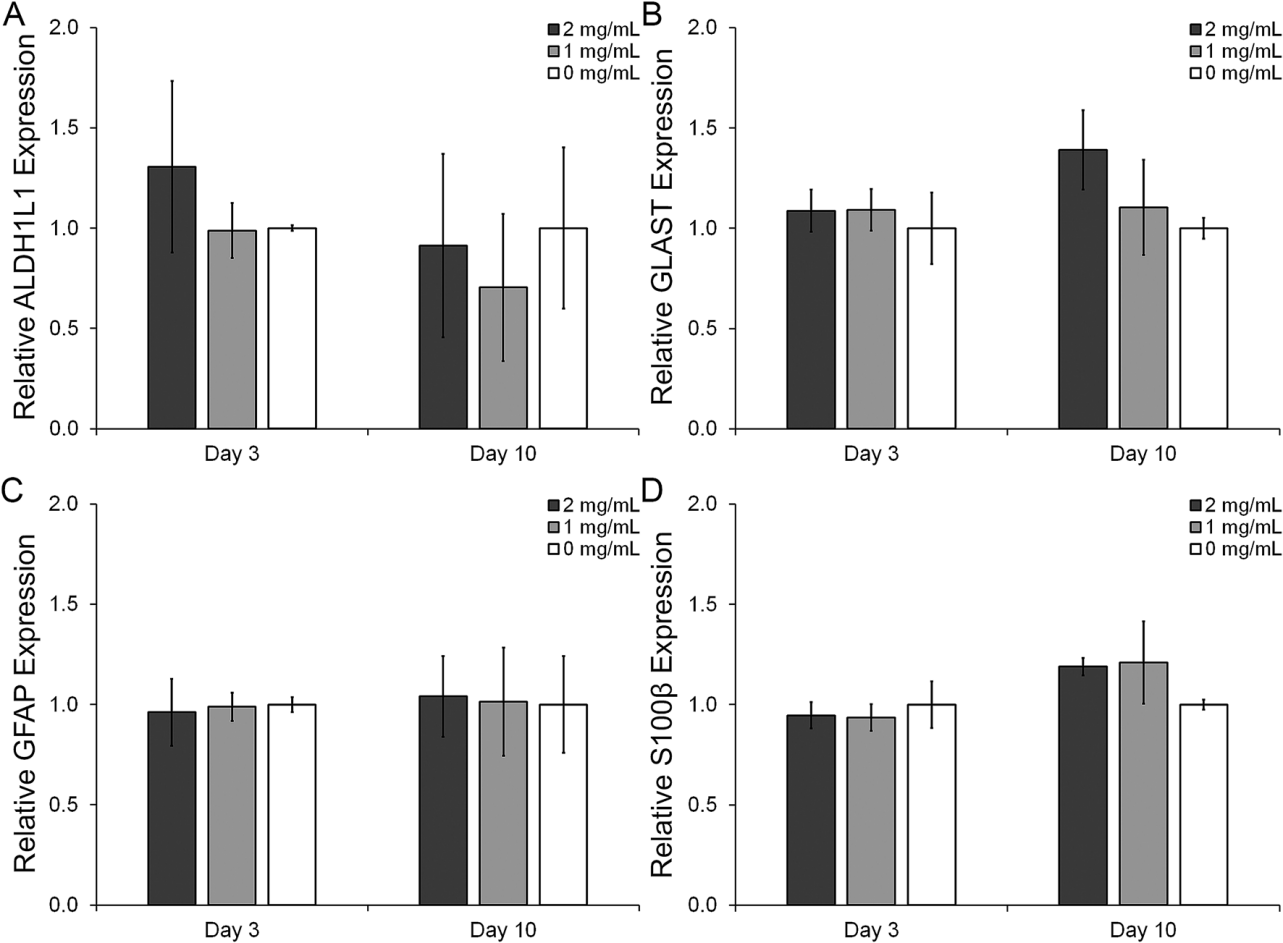
Phenotypical evaluation of human astrocytes using qRT-PCR. Relative expression of ALDH1L1 (**A**), relative expression of GLAST (**B**), relative expression of GFAP (**C**), and relative expression of S100β (**D**).

Astrocytes, a fundamental CNS cell type, are characterized by the relative expression of several proteins which define their phenotype. Among others markers quiescent HAf express relatively high levels of ALDH1L1 and GLAST while protein expression of GFAP and S100β are upregulated in reactive astrocytes. Additionally, markers such as iNOS, IL-1β and TNFα are also associated with reactive astrocytes and contribute to the inflammatory response in CNS tissue^51,52^. To study the effects of the reduction in HA content on astrocyte phenotype, samples from HAf in the mIPNs containing 2, 1 and 0 mg/mL of HA were collected after 3 and 10 days in culture. For each time point qRT-PCR and immunostaining analyses were performed targeting the quiescent markers ADLH1L1 (Fig. 4A) and GLAST (Fig. 4B), and the reactive markers GFAP (Fig. 4C) and S100β (Fig. 4D). The mRNA levels for the inflammatory markers iNOS, IL-1β and TNFα were below the detection limit. To prevent cell mediated contraction in the mIPN scaffolds during the cell spreading, a low cell density must be used. This experimental design limit may have restricted our ability to reach the appropriate levels of mRNA to accurately measure the relative expression of the selected inflammatory markers at the gene expression level.

At day 3, qRT-PCR results show approximately a 23.4% and 24.4% decrease in the expression of the quiescent marker ADLH1L1 for the 0 mg/mL HA and 1 mg/ml HA mIPNs formulations relative to 2 mg/ml HA group. However, differences in mean values across these groups are not statistically significant (p ≥ 0.353). Similarly, the expression of GLAST at day 10 seems to progressively decrease as HA content is reduced in the mIPNs. Relative to the 2 mg/mL HA mIPN, the expression of GLAST was reduced by 20.6% and 28.0% in the 1 mg/mL HA and 0 mg/mL HA mIPNs respectively. Differences across groups were not statistically significant (p ≥ 0.063). In terms of the expression of the reactive markers GFAP and S100β, no significant changes in the mean value were observed after 3 or 10 days in culture (p ≥ 0.109). Cumulatively, the qRT-PCR data appears to suggest that the reduction in HA modulates the HAf phenotype contributing to the downregulation of quiescent marker expression.

**Figure 5:**
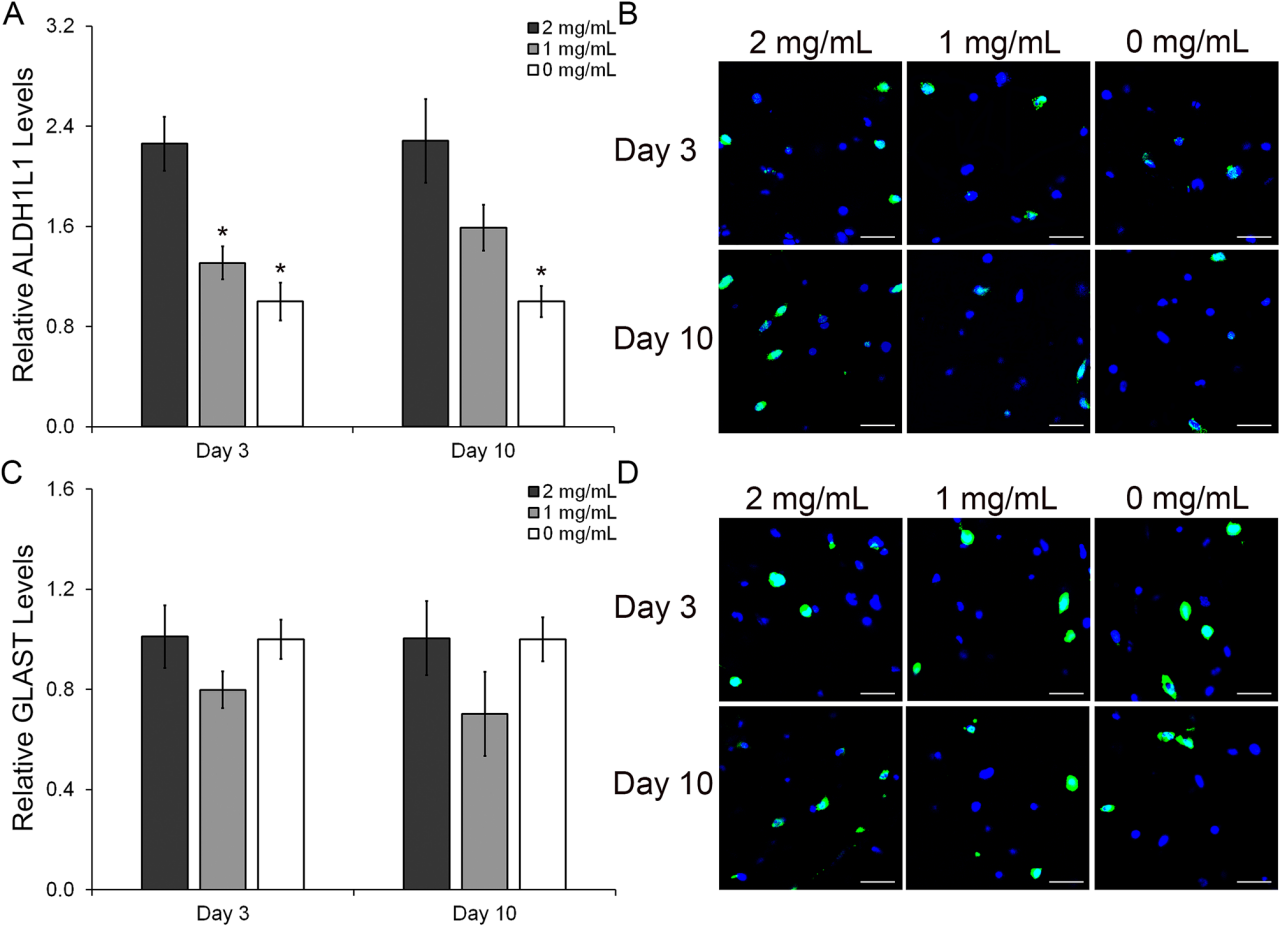
Semi-quantitative analysis of the immunostaining results for the protein expression of the HAf quiescent markers ALDH1L1 and GLAST (**A**,**C**). Representative immunofluorescent images of the ALDH1L1 and GLAST staining (**B**,**D**). The scale bar represents a distance of 100 µm. *Significantly different from 2 mg/mL HA, p < 0.05.

To complement the qRT-PCR results, protein analyses using immunostaining were performed. In contrast to the qRT-PCR results, immunostaining results indicate a significant impact of HA content on the modulation of HAf phenotype at the protein expression level. As shown in Fig. 5A,B at day 3, the relative expression levels of the astrocyte marker ALDH1L1 were reduced by 1.3-fold (p = 0.001) in the 1 mg/mL HA mIPN and by 2.3-fold (p ≤ 0.001) in the 0 mg/mL HA mIPN relative to the 2 mg/mL HA scaffold. At day 10, ALDH1L1 expression was also reduced with the decrease in HA concentration. The levels of ALDH1L1 in the 1 mg/mL HA formulation were reduced 1.4-fold relative to the 2 mg/mL HA mIPN group (p = 0.090) while ALDH1L1 expression in the 0 mg/mL HA mIPN was significantly reduced by a 2.3-fold factor relative to the high HA content group (p = 0.001). In terms of GLAST expression (Fig. 5C,D), relative levels across experimental groups were indistinguishable at either day 3 (p ≥ 0.211) or day 10 (p ≥ 0.147) time points.

**Figure 6:**
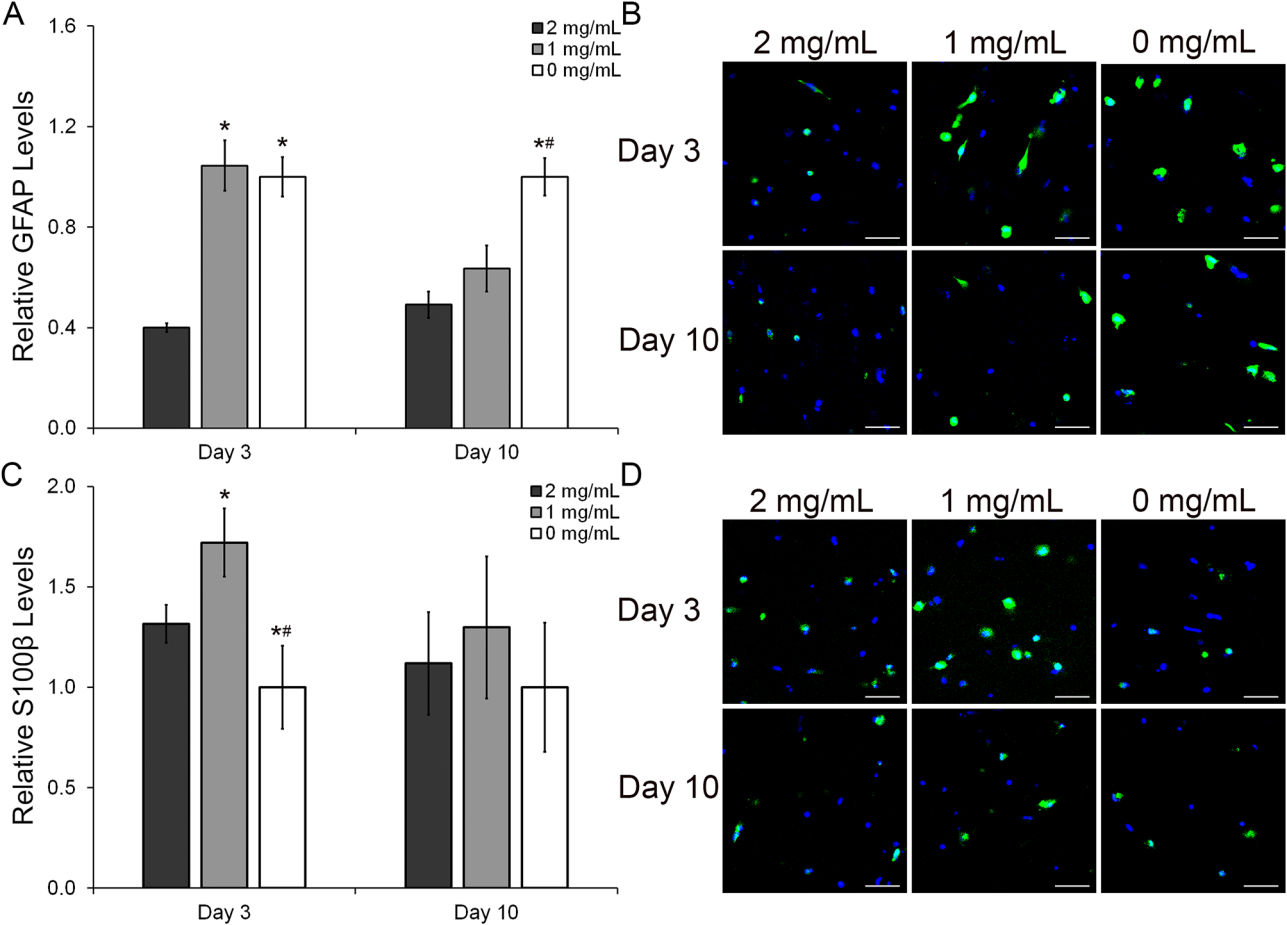
Semi-quantitative analysis of the immunostaining results for the protein expression of the HAf reactive markers GFAP and S100β (**A**,**C**). Representative immunofluorescent images of the GFAP and S100β staining (**B**,**D**). The scale bar represents a distance of 100 µm. *Significantly different from 2 mg/mL HA, p < 0.05: ^#^Significantly different from 1 mg/mL HA, p < 0.05.

In addition to the evaluation of the quiescent markets ALDH1L1 and GLAST, the expression of the reactive markers GFAP and S100β were also evaluated at the protein level. The relative levels of GFAP were increased with the reduction in HA (Fig. 6A,B). The GFAP levels at day 3 (Fig. 6A) were 2.6 and 2.5-fold higher in the 1 mg/mL (p < 0.001) and 0 mg/mL (p < 0.001) relative to 2 mg/mL HA mIPNs. After 3 days in culture, differences between the mean GFAP levels in the 1 mg/mL HA and 2 mg/mL HA mIPNs were not observed. At day 10, there is a progressive increase in the levels of GFAP corresponding to the decreasing HA content. However, significant differences in mean values were only observed between the 0 mg/mL HA and the 2 mg/mL HA mIPNs. The GFAP expression in the 0 mg/mL HA mIPN was increased by 2.0 and 1.6-fold relative to 2 mg/mL HA (p < 0.000) and 1 mg/mL HA (p = 0.004) formulations respectively. Our results and observations for the relative expression of GFAP as a function of HA content are in agreement with previously reported literature^53,54^. The expression of the reactive marker S100β showed a very dynamic pattern (Fig. 6C). At day 3, S100β levels were 1.3-fold and 1.7-fold higher in the 1 mg/mL HA formulation relative to the 2 mg/mL HA mIPN and the 0 mg/mL HA mIPN (p ≤ 0.019). Moreover, HAf in the 0 mg/mL HA mIPN exhibited 1.3-fold and 1.7- fold lower S100β levels relative to the other two experimental groups (p ≤ 0.019). Differences across groups in S100β initially observed at the early time point disappeared after 10 days in culture. Mean values across sample groups for the S100β levels after 10 days in culture were statistically indistinguishable (p ≥ 0.174).

**Figure 7:**
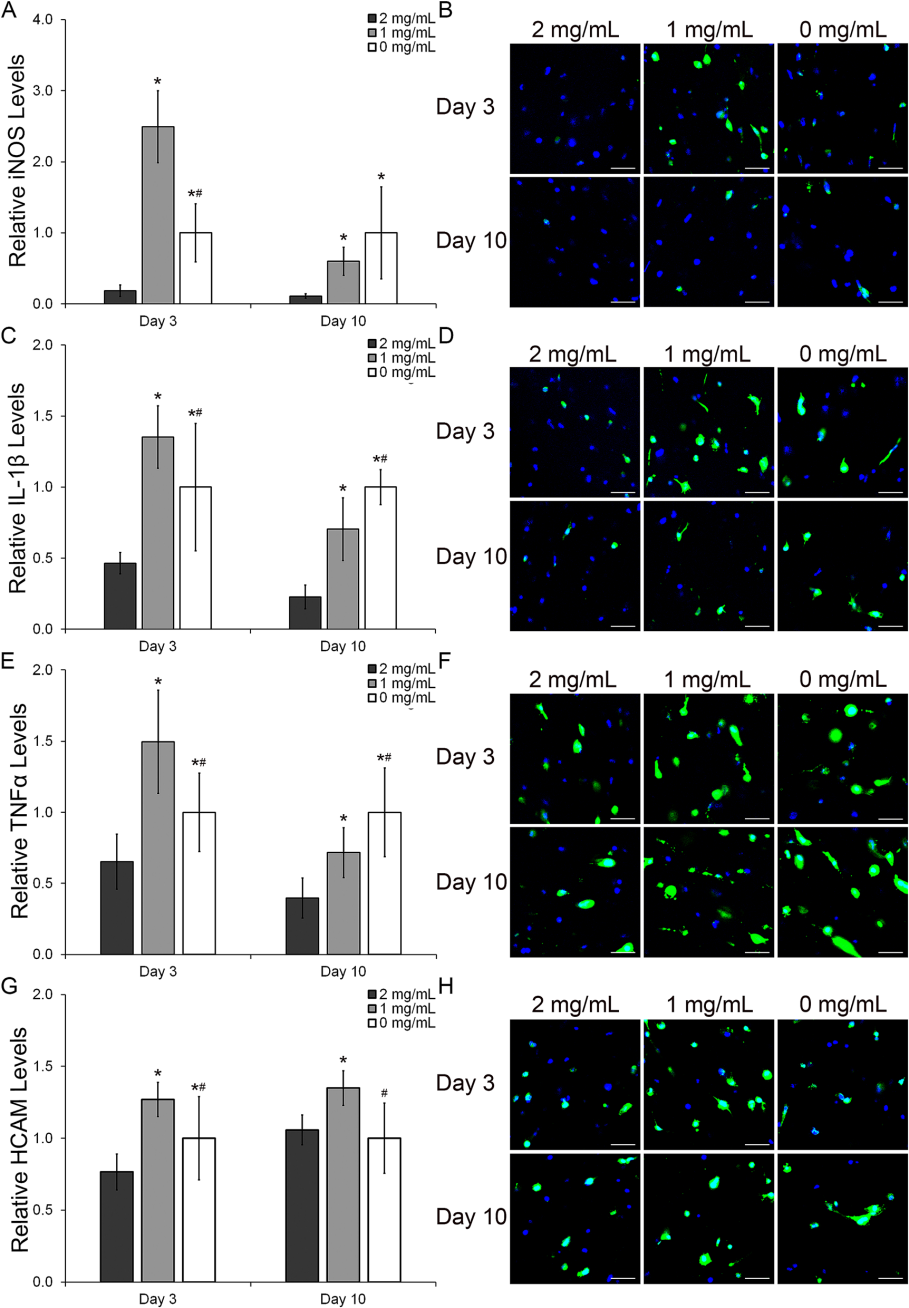
Semi-quantitative analysis of the immunostaining results for the protein expression of the HAf inflammatory markers iNOS, IL-1β, TNFα and the cell receptor HCAM (**A**,**C**,**E**,**G**). Representative immunofluorescent images of the iNOS, IL-1β, TNFα and the cell receptor HCAM staining (**B**,**D**,**F**,**H**). The scale bar represents a distance of 100 µm. *Significantly different from 2 mg/mL HA, p < 0.05: ^#^ Significantly different from 1 mg/mL HA, p < 0.05.

Astrocytes also contribute the inflammatory response in CNS tissue. To evaluate the effects of the reduction in HA on the inflammatory response, HAf were stained for 3 inflammatory markers: iNOS (Fig. 7A,B), IL-1β (Fig. 7C,D) and TNFα (Fig. 7E,F). These markers are key regulatory cytokines in the inflammatory response of human tissue^51,52^. As it is shown in Fig. 7 the trend for the inflammatory response at each time point was very similar for all three markers. After 3 days in culture, HAf in the 1 mg/mL HA mIPN exhibited higher levels of iNOS, IL-1β and TNFα relative to the other two formulations. Relative to the 2 mg/mL HA mIPN formulation, the protein levels in the 1 mg/mL HA mIPN were increased by 13.4-fold, 2.9-fold and 2.3-fold (p < 0.001) for iNOS, IL-1β and TNFα respectively. Similarly, the expression of iNOS, IL-1β and TNFα was 2.5-fold, 1.4-fold and 1.5-fold higher in the 1 mg/mL HA group when compared with the levels of the inflammatory markers in the 0 mg/mL HA mIPN (p ≤ 0.005). In comparison with day 3 results, at day 10, the levels of the three inflammatory markers increased progressively with the decrease in HA content (Fig. 7A,C,E). The expression of iNOS (Fig. 7A) in the 0 mg/mL HA group was 9.0-fold and 1.7-fold higher than in the 2 mg/mL (p < 0.001) and 1 mg/mL HA mIPNs (p = 0.079) respectively. Furthermore, the levels of iNOS in the 1 mg/mL HA formulation were significantly elevated relative to 2 mg/mL mIPN (p = 0.043). In terms of IL-1β expression, its normalized levels (Fig. 7C) were increased from 0.22 in the 2 mg/mL HA formulation to 0.7 and 1.0 in the 1 mg/mL HA and 0 mg/mL HA mIPNs respectively (p < 0.001). Finally, a similar trend was observed for the expression of TNFα (Fig. 7E) which was increased by a factor of 1.4 for 1 mg/mL HA and 2.5 factor for 0 mg/mL HA mIPNs when compared with the 2 mg/mL HA experimental group (p ≤ 0.001). We hypothesize that the increase in culture time, accentuates the differences in the inflammatory response among groups in the day 10 relative to the day 3 time point. The initial stress of the cells during the encapsulation process could have also impacted the expression of the inflammatory markers at day 3. Cumulatively, the expression of inflammatory cytokines in HAf is enhanced with the reduction in HA in the proposed engineered scaffolds. The upregulation of these inflammatory markers have been previously observed in astrocytes exposed to the presence Aβ^55^. These findings are very significant since they suggest that the reduction in HA content in the absence of changes in scaffold modulus elicits an inflammatory response similar to that in astrocytes exposed to key biochemical molecules associated with the progression of neurodegenerative diseases.

To gain insight on a possible mechanism regulating astrocyte response to different levels of HA-HMW, the relative expression of the cell receptor HCAM was evaluated at the protein expression level (Fig. 7G,H). The upregulation of this cell marker has been associated with astrogliosis and the increase in the inflammatory response of astrocytes^56–58^. Furthermore, high expression of HCAM has been observed in astrocytes from Alzheimer patients^59^. In our model, we observed that after 3 days in culture and relative to the 2 mg/mL HA group, the expression of HCAM was 1.7-fold and 1.3-fold higher in the 1 mg/mL and 0 mg/mL HA formulation respectively, these differences were statistically significant (p ≤ 0.010). Moreover, the expression of HCAM in the 1 mg/mL group was 1.3-fold higher than in the 0 mg/mL HA formulation (p = 0.002). The trend for the relative expression of this marker at day 3 follows closely the trend observed for the expression of the selected inflammatory markers. This observation indicates that perhaps initially, HCAM is the primary receptor involved in the inflammatory signaling pathway in our model. After 10 days in culture, the expression of HCAM in the 1 mg/mL formulation is 1.3-fold higher relative to the other two groups. Relative to the 2 mg/mL HA and 0 mg/mL HA the p values for the comparison were p = 0.001 and p < 0.001 respectively. Differences in the expression of this receptor between the 2 mg/mL HA and the 0 mg/mL were not statistically significant (p = 0.684). Since HA can also interact with other receptors including the Toll-Like Receptors 2 and 4 (TLR-2, TLR-4), and the Hyaluronan-Mediated Motility Receptor (HMMR) it is possible that the inflammatory signaling depends on multiple routs and that different HA receptors may be activated at different time points. Given the current information we cannot clearly present a possible controlling signaling pathway for the biological responses of human astrocyte cultured in our proposed mIPN based platform.

## Conclusions

In this work we demonstrated that the proposed mIPN platform can be used to study the effects of compositional changes in HA levels on HAf morphology and phenotype. We specifically illustrated that in the absence of significant changes in mechanical performance of the scaffolds, the decrease in HA concentration—which is normally associated with tissue degradation—is a significant contributing factor for an increase in the reactive and inflammatory response of human astrocytes. Overall, the reduction in HA content contributes to the decrease in total processes length of human astrocytes at least within the range of explored HA-HMW concentrations and experimental conditions. Furthermore, the expression of quiescent markers is attenuated as the HA concentration in the scaffolds is reduced while the expression of reactive and inflammatory markers is significantly upregulated. We recognize that the current work does not address all variables involves in the degradation process of HA. However, this work demonstrates that our mIPN platform has the potential to be tuned to explore the effects of different HA molecular weights and the presence of small soluble HA fragments on the biological response of human astrocytes while maintaining mechanical performance similar to those in native tissue. We also believe that in the future, this family of scaffolds could be potentially used as a human tissue disease model to evaluate effectiveness of therapeutic molecules on the regulation of the inflammatory responses of human astrocytes in 3D contexts.

## Supplementary information

Supplementary Information 1.

Supplementary Information 2.

Supplementary Information 3.

Supplementary Information 4.
