# Supplementary material for: Modeling the effects of hyaluronic acid degradation on the regulation of human astrocyte phenotype using multicomponent interpenetrating polymer networks (mIPNs)
Source: Sci Rep. 2020 Nov 26;10:20734. doi: 10.1038/s41598-020-77655-1 (PMC7691997; doi:10.1038/s41598-020-77655-1)
Supplement: Supplementary file 1 — Supplementary Information 1. [file 41598_2020_77655_MOESM1_ESM.pdf]

Modeling the Effects of Hyaluronic Acid Degradation on the Regulation of Human Astrocyte Phenotype Using Multicomponent Interpenetrating Polymer Networks (mIPNs)

Andrea C. Jimenez-Vergara[1], Rachel Van Drunen[2], Tyler Cagle[2], and Dany J. Munoz-Pinto[1,2*]

[1] Engineering Science Department, Trinity University, San Antonio, TX 78212, United States

[2] Neuroscience Program, Trinity University, San Antonio, TX 78212, United States

***Corresponding author**:
Department of Engineering Science, Neuroscience Program
Center for the Sciences and Innovation, CSI 470C
Trinity University
One Trinity Place
San Antonio, TX 78212
Tel: 1-(210)-999-7565
Fax: 1-(210)-999-8037
E-mail: dmunozpi@trinity.edu

Keywords: Human astrocytes, interpenetrating polymer networks, and hyaluronic acid.

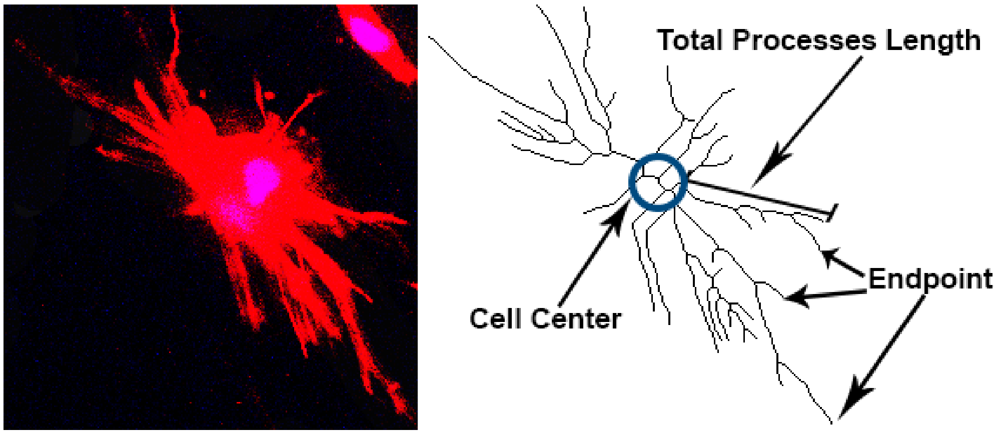

Supplementary Figure 1. Example images of an astrocyte cell and its corresponding skeleton image. The skeleton image was generated using ImageJ.
