# Supplementary material for: Modeling the effects of hyaluronic acid degradation on the regulation of human astrocyte phenotype using multicomponent interpenetrating polymer networks (mIPNs)
Source: Sci Rep. 2020 Nov 26;10:20734. doi: 10.1038/s41598-020-77655-1 (PMC7691997; doi:10.1038/s41598-020-77655-1)
Supplement: Supplementary file 4 — Supplementary Information 4. [file 41598_2020_77655_MOESM4_ESM.pdf]

Modeling the Effects of Hyaluronic Acid Degradation on the Regulation of Human Astrocyte Phenotype Using Multicomponent Interpenetrating Polymer Networks (mIPNs)

Andrea C. Jimenez-Vergara[1], Rachel Van Drunen[2], Tyler Cagle[2], and Dany J. Munoz-Pinto[1,2*]

[1] Engineering Science Department, Trinity University, San Antonio, TX 78212, United States

[2] Neuroscience Program, Trinity University, San Antonio, TX 78212, United States

***Corresponding author**:
Department of Engineering Science, Neuroscience Program
Center for the Sciences and Innovation, CSI 470C
Trinity University
One Trinity Place
San Antonio, TX 78212
Tel: 1-(210)-999-7565
Fax: 1-(210)-999-8037
E-mail: dmunozpi@trinity.edu

Keywords: Human astrocytes, interpenetrating polymer networks, and hyaluronic acid.

Supplementary Table 2. Antibodies List.

Antibody	Clone	Manufacturer
GLAST	A3	Santa Cruz Biotechnology
ALDH1L1	YY8	Santa Cruz Biotechnology
GFAP	2E1	Santa Cruz Biotechnology
S100β	9A11B9	Santa Cruz Biotechnology
iNOS	C-11	Santa Cruz Biotechnology
IL-1β	E7-2-hIL1β	Santa Cruz Biotechnology
TNFα	C-4	Santa Cruz Biotechnology
HCAM	F4	Santa Cruz Biotechnology
